# Genome-Wide Copy Number Variation Analysis in Extended Families and Unrelated Individuals Characterized for Musical Aptitude and Creativity in Music

**DOI:** 10.1371/journal.pone.0056356

**Published:** 2013-02-27

**Authors:** Liisa Ukkola-Vuoti, Chakravarthi Kanduri, Jaana Oikkonen, Gemma Buck, Christine Blancher, Pirre Raijas, Kai Karma, Harri Lähdesmäki, Irma Järvelä

**Affiliations:** 1 Department of Medical Genetics, University of Helsinki, University of Helsinki, Finland; 2 Department of Information and Computer Sciences Aalto University, Espoo, Finland; 3 Wellcome Trust Centre for Human Genetics, Oxford, United Kingdom; 4 Sibelius Academy, DocMus Department, Helsinki, Finland; 5 Sibelius Academy, Department of Music Education, Helsinki, Finland; Yale School of Public Health, United States of America

## Abstract

Music perception and practice represent complex cognitive functions of the human brain. Recently, evidence for the molecular genetic background of music related phenotypes has been obtained. In order to further elucidate the molecular background of musical phenotypes we analyzed genome wide copy number variations (CNVs) in five extended pedigrees and in 172 unrelated subjects characterized for musical aptitude and creative functions in music. Musical aptitude was defined by combination of the scores of three music tests (COMB scores): auditory structuring ability, Seashores test for pitch and for time. Data on creativity in music (herein composing, improvising and/or arranging music) was surveyed using a web-based questionnaire.

Several CNVRs containing genes that affect neurodevelopment, learning and memory were detected. A deletion at 5q31.1 covering the protocadherin-α gene cluster (*Pcdha 1-9*) was found co-segregating with low music test scores (COMB) in both sample sets. *Pcdha* is involved in neural migration, differentiation and synaptogenesis. Creativity in music was found to co-segregate with a duplication covering glucose mutarotase gene *(GALM)* at 2p22. *GALM* has influence on serotonin release and membrane trafficking of the human serotonin transporter. Interestingly, genes related to serotonergic systems have been shown to associate not only with psychiatric disorders but also with creativity and music perception. Both, *Pcdha* and *GALM*, are related to the serotonergic systems influencing cognitive and motor functions, important for music perception and practice. Finally, a 1.3 Mb duplication was identified in a subject with low COMB scores in the region previously linked with absolute pitch (AP) at 8q24. No differences in the CNV burden was detected among the high/low music test scores or creative/non-creative groups. In summary, CNVs and genes found in this study are related to cognitive functions. Our result suggests new candidate genes for music perception related traits and supports the previous results from AP study.

## Introduction

Like intelligence or language, music perception and practice are complex cognitive functions of the human brain. Twin studies have shown evidence for genetic overlap between different cognitive abilities such as learning, reading and mathematics [1]. Neurobiological studies [2], [3], [4], [5], studies on animals [6], [7], [8], and human infants [9], [10], [11], [12] have provided evidence on biological basis of music perception. There is an abundance of data about the neurophysiological effects of music perception and practice on the human brain [2], [3], [4], [5], [12]. However data on the effects at the cellular is so far missing.

Recent studies have shown a substantial genetic component in music perception including absolute pitch [13], congenital amusia [14], auditory structuring ability [15], [16], [17] and musical ability [18]. Until now, little evidence for the molecular genetic background of musical aptitude has been obtained. In the absence of such evidence, we and others have performed both genome wide analyses and candidate gene studies in musical traits [13], [15], [16], [17], [18], [19], [20]. Intriguingly, genome-wide analyses performed separately in Finnish and Mongolian populations with different music phenotypes (musical aptitude and musical ability) revealed linkage in the partly overlapping genetic regions at chromosome 4q [15], [18]. Previously, such candidate genes as *AVPR1A*, *SLC6A4*, *UNC5C* and *UGT8* have been suggested for musical abilities [15], [16], [17], [18]. These preliminary molecular studies support the hypothesis that musical aptitude is the result of currently unknown number of genomic variations, environment, and their complex interactions.

Variations in the human genome range from large chromosomal anomalies (size >2–5 Mb) to single nucleotide polymorphisms (SNPs) (size from 1 to 700 bp) [21]. Copy number variations (CNVs) are structural genomic variants arising from deletions or duplications of the genomic region. CNVs show high variability in the human genome and have been suggested to have multiple effects on gene function, evolution and disease risk [22], [23]. To date, CNVs have been shown to have an important role in cognitive function of the human brain in neuropsychiatric disorders [23], [24], [25], [26] and in common complex diseases [27], [28]. Lately, attention has been paid to CNVs underlying normal human traits like height and intelligence [29], [30], [31]. Most of these studies have been performed in case-control settings and have focused on diseases [32] whereas family-based studies on normal cognitive traits are rare.

In order to obtain further understanding about the biological basis of music perception, we conducted a genome-wide survey of CNVs in five multigenerational families and in 172 unrelated subjects who were characterized for musical aptitude and creativity in music. The advantages of a family-based setting used here over population-based include better control of population stratification, enrichment of rare variants and the ability to discriminate variants co-segregating with the trait [33], [34]. High mutation rate is resulting in a large number of *de novo* variations [23]. The family based approach is useful as it offers validation for CNV each time it is inherited.

## Materials and Methods

### Ethics statement

The study was approved by The Ethical Committee of Helsinki University Central Hospital and was conducted in accordance with the Declaration of Helsinki. An informed consent was obtained from all subjects.

### Study material

The age of the participants varied from 18 to 60 years. To minimize the age-related affects on CNV accumulation and various music tests, we made sure that all the individuals participating in this study were aged ≤60 years.

**Figure 1:**
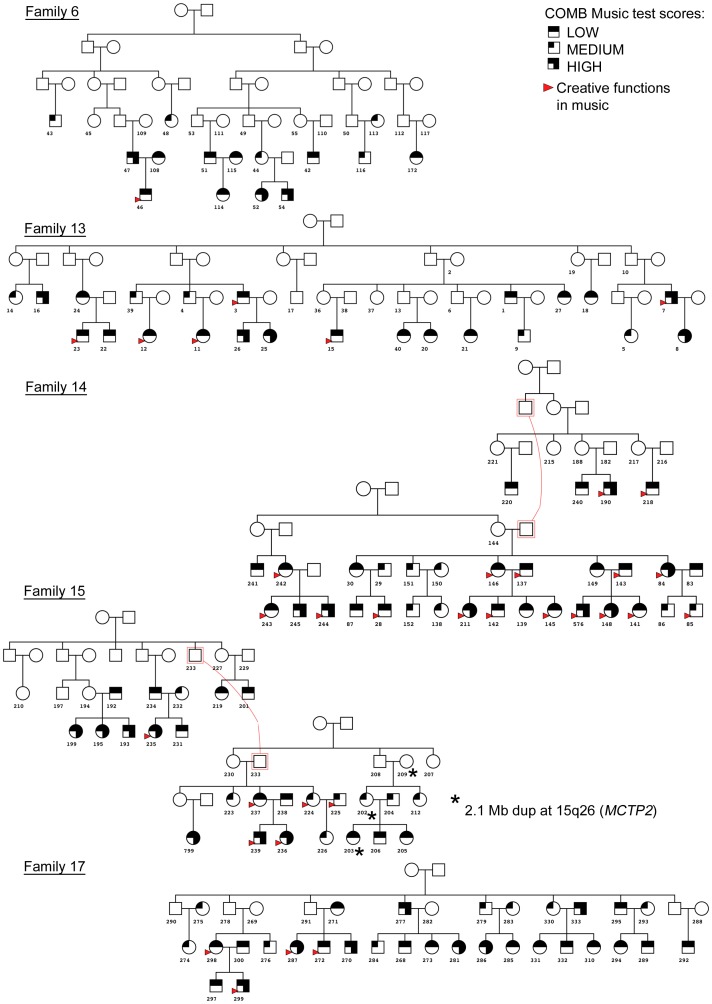
The families of the study. Circles represent females, squares males and genotyped individuals were marked with subject numbers.

The family pedigrees are shown in Figure 1. The families consist of from 28 to 38 genotyped members whose combined music test scores (COMB) and creative functions in music are known (Table 1).The families participate in the study where genetic background of music perception and practice is studied [15], [16], [17]. Of the study families, family 6 has the lowest mean COMB scores (116.98), and creative functions in music were reported by 10%. Family 14 has the highest mean COMB scores (128.21) and 46% of the members reported creative functions in music.

**Table 1:** Characteristics of each family and the unrelated sample set (N-value).

Pedigree no.		6	13	14	15	17	unrelated
**DNA available**		25	32	39	36	38	172
**sex**	**male**	12 (48%)	16 (50%)	20 (53%)	15 (42%)	18 (47%)	71 (41%)
	**female**	13 (52%)	16 (50%)	18 (47%)	21 (58%)	20 (53%)	101 (59%)
**COMB music test scores**	**mean**	116.98	120.66	128.21	117.17	118.3	122.41
	**range**	93.5–144.25	88.25–141.5	82–147	83–147	90–144.8	89–148
**Creativity in music**	**No**	18 (90%)	19 (70%)	19 (54%)	22 (79%)	30 (88%)	125 (86%)
	**Yes**	2 (10%)	8 (30%)	16 (46%)	6 (21%)	4 (12%)	21 (14%)

Further, a sample set of 172 unrelated subjects were selected to analyze the effect of CNV burden and the enrichment of CNVs in opposite phenotypes of musical aptitude and creative functions in music (Table 1). The unrelated subjects originate from the genetically isolated Finnish population that has experienced multiple bottlenecks in its population history [35] so we cannot fully exclude their distant relatedness. In the questionnaire participants could report if any close relatives participated in the study. The individuals in the unrelated sample set were selected if that they did not report any relatives in the study or did not show relatedness in identity by descent (IBD) analysis. No medical information was available from the participants but as far as we know, they are healthy. The recruitment process has been described in our earlier studies [15], [16], [17].

### Phenotypes

Musical aptitude of each participant was defined using three music tests: the auditory structuring ability test (Karma Music test, KMT) [36] and Carl Seashore's pitch and time discrimination subtests (SP and ST respectively) [37] described previously by Pulli et al. [15] and Ukkola et al. [16]. Shortly, KMT contains 40 items that measure recognition of melodic contour, grouping, relational pitch processing, and gestalt principles, the same potentially innate musical cognitive operations reported by Justus & Hutsler [38]. In contrast, Seashore's tests each contain 50 items that measure simple sensory capacities, such as the ability to detect small differences in tone pitch or duration that are necessary in music perception. A combined music test score (COMB), was computed as the sum of the separate scores of the three individual test results (range from 75 to 150 scores), as described earlier [16], [17]. There is moderate correlation between the three music tests scores: 0.61 between KMT and SP, 0.42 between KMT and ST and 0.38 between SP and ST (P<0.0001 for all three) [17]. The SP has shown the highest heritability (52%) whereas the heritability estimates for KMT, ST and COMB are 39%, 10% and 44% respectively [16], [17]. The reliabilities of the music tests range from 0.78 to 0.91. [15]. The age had a significant effect on COMB scores (F = 19.24, df = 169, p = 2.02*10^−5^). The R-square of 0.09 suggests that about 9% of the variability in COMB scores could be explained by age.

Background information, e.g. about the participant's creativity in music was collected using a web-based self-report questionnaire as described earlier [16], [17]. Here the creative functions in music were defined as having one or several of the following: composing, improvising or arranging music. The questionnaire is available from the authors on request.

In this study, the music phenotypes were explored in opposite phenotypes. Technically, we compared the distribution of CNVs among subjects belonging to the two groups of COMB scores. Based on the lower and upper quartiles of COMB scores, the subjects were divided into (1) low and (2) high COMB scoring group within each family and also unrelated sample (Table 2) (3) “Creative phenotype” here means a subject reporting one or several of the aforementioned creative functions in music and (4) “noncreative phenotype” was applied if not reporting any of these activities. Data about creativity was available in family 6 from 48%, family 13 from 63%, family 14 from 75%, family 15 from 53%, family 17 from 74% and unrelated data from 73% of the family members participating in the study (Table 2).

**Table 2:** Descriptive statistics for the analyzed music phenotypes in the study material.

		COMB scores		Creative phenotype	
		High (% of total N)	Low (% of total N)	Yes (% of total N)	No (% of total N)
**Fam no. 6**		>144.2	<115.0		
	**N**	5 (20%)	4 (16%)	1 (4%)	11 (44%)
**Fam no. 13**		>138.8	<114.5		
	**N**	5 (16%)	5 (16%)	6 (19%)	14 (44%)
**Fam no. 14**		>141.8	128.5		
	**N**	7 (18%)	7 (18%)	17 (44%)	12 (31%)
**Fam no. 15**		>137.0	<105.0		
	**N**	6 (17%)	6 (17%)	5 (14%)	14 (39%)
**Fam no. 17**		>133.2	<111.5		
	**N**	8 (21%)	8 (21%)	4 (11%)	24 (63%)
**Unrelated**		>138.2	<116.2		
	**N**	40 (23%)	28 (16%)	18 (10%)	108 (63%)

### Genotyping

Peripheral blood samples for genomic DNA extraction were drawn from the participants over 12 years of age (no cell lines were used). 200 ng of DNA from each subject was genotyped using the Illumina Infinium Human OmniExpress-12v1 beadchip, which contains an approximate number of 733,202 markers per sample for SNP and CNV analyses. Samples were genotyped with an average overall call rate of 99.54%. Normalized signal intensity data was obtained through Illumina BeadStudio software. Information on Log_2_ R ratios, B allele frequencies, markers and chromosomal coordinates from each sample were used for CNV identification. This is the first study using the SNP data obtained from the analysis.

### CNV identification and quality control

We mapped all the probe coordinates in this study to human genome build GRCh37/hg19. CNVs were identified using two Hidden Markov Model (HMM) -based algorithms: PennCNV and QuantiSNP. At present, both of these algorithms constitute the most reliable set-up for CNV detection using Illumina platforms [39]. Additionally, this multi-algorithm approach increases the confidence of CNV calls and reduces false positives [39], [40], [41]. For familial data, using the tro-based CNV calling in PennCNV, we validated the inheritance status of CNVs. Further, to ensure good quality of the data, we followed stringent quality control criteria at both sample-level and CNV-call level. Specifically, we categorized certain samples as outliers and eliminated them if they meet any of the following criteria: (a) Call rate below the call threshold of 98%. (b) Standard deviation of Log_2_ R ratio more than 0.15 (c) Standard deviation of B allele frequency more than 0.05 (d) Waviness factor outside the limits of −0.04 and 0.04 (4) BAF drift more than 0.002 (e) Number of detected CNVs per sample more than 50. At the level of CNV-calls, CNVs identified using both PennCNV and QuantiSNP were retained only if their size was larger than 10 bp and their log Bayesian factor was more than 10 respectively. A CNV detected by both algorithms was merged into a single consistent-call (consensus) by using the outermost boundaries defined by either of the algorithm, irrespective of their size of overlap. We retained only such consistent calls for further analyses.

### Statistical analyses

To unravel the impact of CNVs on musical aptitude and creative functions in music, we analyzed the detected CNVs using three different approaches: First, we analyzed the inheritance of CNVs in extended pedigrees and their penetrance in contrasting phenotypes, i.e. high COMB vs low COMB and creative vs non-creative subjects. The main objective of this analysis is to find the highly penetrant CNVs that are private to a specific phenotype. For this, CNVRs larger than 10 kb were ranked in each family in individuals of contrasting phenotypes (Table 2). This straightforward ranking method ranks a CNV based on its frequency count in the “affected” individuals of each family. For example, the CNV that appears the most frequent in musically creative individuals of a family is given a higher rank for musical creativity within that family. This ranking method was described earlier in Karlsson et al. [26]. Second, we investigated the effect of CNV burden on 172 unrelated individuals characterized for the aforementioned phenotypes. For this, we tested if there was an increased burden of CNVs in opposite phenotypes concerning number of CNVs and CNV size using a two-sided Fisher's exact test. Third, we checked if any particular CNV was present with increased frequency in the opposite phenotypes of unrelated individuals using a two-sided Fisher's exact test; p-value<0.05. All these Statistical analyses were performed using R (statistical computing environment, http://www.r-project.org/), SPSS predictive analytics software version 20, PLINK and custom scripts.

## Results

### CNVRs in musical aptitude (COMB scores)

In the five study families all CNVRs were ranked for COMB scores and are shown in Supplementary material (Table S1). High ranking CNVRs shared by individuals in at least two families for the COMB scores are shown in Table 3. One CNVR was ranked high in high COMB scores. A total of 67% of family members with high COMB scores in families 6 and 14 members carried a deletion at 1q21.2 (Table 3). The region contains genes *FCGR1C* and *LOC388692* and has previously been linked with neurodevelopmental disorders (schizophrenia, autism, ADHD, mental retardation, learning disabilities and dyslexia) [42], [43]. A family-specific deletion at 8p23 (*DLGAP2*), previously related to autism [39], was also ranked high in family 14.

**Table 3:** High-ranked CNVRs for COMB scores and creative phenotype in the analyzed families.

	freq. in the phenotype	Chr region	Chr: start-end	Event type	Genes	Families
**High COMB music test scores**	67%	1q21.2	1:149039031–149388389	Loss	*FCGR1C, LOC388692*	6, 14
**Low COMB music test scores**	54%	5q31.3	5:140225908–140237548	Loss	*Protocadherin alpha gene cluster*	14, 15
**Creative phenotype**	48%	5p15.33	5:788646–840717	Loss	*ZDHHC11*	14, 17
	27%	2p22.1	2:38955129–38977612	Gain	*GALM*	14, 15
	24%	10q11.22	10:47412588–47703869	Gain	*ANTXRL*	14, 17
	20%	3p22.2	3:37979882–37986249	Loss	*CTDSPL*	13,17
	19%	5p15.31	5:8258881–8260630	Loss	-	14,17
**Noncreative phenotype**	31%	2p12	2:76941049–76949101	Loss	-	14,17
	21%	3p14.1	3:65191847–65214685	Loss	-	13,17
	19%	3q28	3:191065392–191072060	Loss	*CCDC50*	6, 14, 17

In low COMB scoring individuals one CNVR was ranked high in two different families. This was a deletion at 5q31.3 found in 54% of low COMB scoring individuals in families 14 and 15. The region contains the protocadherin-α gene cluster 1–9 (*Pcdha 1-9*) consisting of 14 tandemly arranged genes [44]. Protocadherins are composed of α-, β- and γ-clusters arranged in tandem on human chromosome 5 and expressed in vertebrate brain [45], [46]. The *Pcdha* encode diverse proteins whose functions are involved in axonal projection and in learning and memory. Several other family-specific interesting CNVRs were ranked high for low COMB scores. In family 14 there was a duplication at 17q21.31 that contains the *KANSL1* (also called *KIAA1267*) gene. Also, a deletion at 9p21.1 (*LINGO1*) in family 15 was ranked high for low COMB scores.

**Figure 2:**
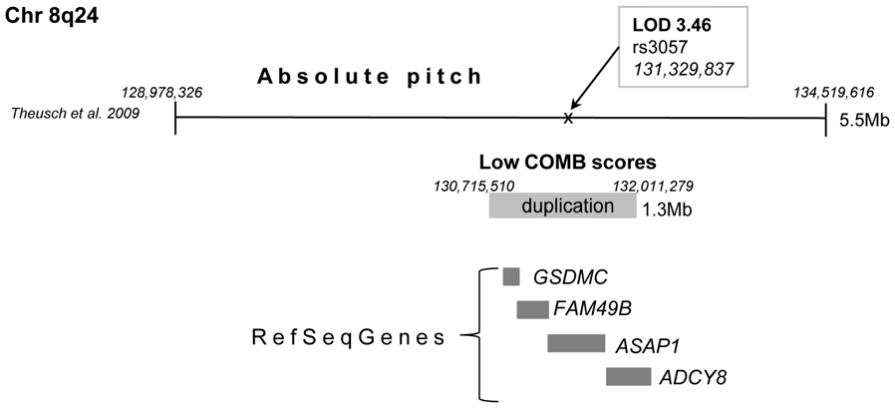
1.3 Mb duplication in the region previously linked with absolute pitch.

A number of large family-specific CNVs were detected in low COMB scoring individuals. A novel 2.1 Mb duplication (not found in the Database of Genomic Variants) at 15q26 was inherited in family 15 in three generations (Figure 1, ID 209, 202, 203). Interestingly, this region contains the *MCTP2* gene previously associated with schizophrenia in samples consisting of Norwegian, Swedish and Danish subjects [47]. In family 6 a novel large 1.3 Mb duplication was identified at 8q24.22 (rs4518624–rs9297816) in a subject with low COMB scores (Figure 1, family 6 ID 48). This region contains the genes *ADCY8*, *ASAP1*, *FAM49B* and *GSDMC*. The duplication is located inside the region that has previously been linked with absolute pitch (rs755520–rs2102861) [13] (Figure 2).

No significant excess of large CNVs or CNV burden were detected in unrelated individuals with high or low COMB scores (Table S2).

### CNVRs in the creative phenotype

In the five study families CNVRs were ranked for creative phenotype and are shown in the Supplementary material (Table S3). A total of five CNVRs were ranked high in the creative phenotype in at least two different families (Table 3). A deletion at 5p15.33 containing the gene *ZCHHC11* was present in 48% and a duplication at 2p22.1 containing the gene *GALM* was present in 27% of creative family members (family 14 and 17; family 14 and 15, respectively). *GALM* encodes galactose mutarotase which is functioning in serotonin metabolism [48], [49]. Curiously, we and others have studied the association of the serotonin transporter (5-HTT) in music related phenotypes [16], [17], [19], [20], [50]. A duplication at 10q11.22 was found in 24% and a deletion at 3p22.2 in 20% of the creative phenotype subjects in two families each (families 14 and 17; families 13 and 17, respectively). 19% of the members in families 14 and 17 carried an inherited deletion at 5p15.31. Family-specific deletions at 6q12 [51] and at 8p23.3 containing the *DLGAP2* gene, a candidate gene for autism [39], were ranked high in creativity (Table S3). The deletion at 8p23.3 was also ranked high among high COMB scores (Table S3).

Three CNVRs were ranked high for noncreative individuals from at least two different families. A deletion at 2p12 was carried by 31% and a deletion at 3p14.1 by 21% of the noncreative subjects in two families (family 14 and 17; family 13 and 17, respectively). 19% of non-creative individuals from three families (6, 14 and 17) carried a deletion at 3q28 containing the *CCDC50* gene.

In the unrelated sample set no significant excess of large CNVs or CNV burden were detected in the creative or non-creative phenotypes (Table S2).

### Association of CNVRs with music phenotypes in the unrelated sample set

After family-based ranking of CNVRs in COMB scores and creative phenotype was completed, the association analysis in the unrelated sample set was performed (Table 4 and Table 5). A duplication at 12p11.21 was slightly enriched in individuals with high COMB scores (Fisher p = 0.0385), while a deletion at 3p14.1 was observed in individuals with low COMB scores (Fisher p = 0.0322). No known genes were located in these CNVRs. The deletion at 3p14.1 was also found in the noncreative family members in CNVR ranking analysis (Table 3). A deletion at 5q31.1 containing *Pcda* was seen in 7% of low COMB and in none of the high COMB subjects.

**Table 4:** Association of CNVRs with music test scores in the unrelated dataset.

	High COMB (N = 40)	Low COMB (N = 28)	Chr region	Chr: start-end	Event type	Genes	p-value
**COMB music test scores**	8 (20%)	13 (46%)	3p14.1	3: 65191847–65214685	loss	-	0.0322
	6 (15%)	0 (0%)	12p11.21	12: 31266287–31409778	gain	-	0.0385

**Table 5:** Association of CNVRs with creative functions in music in the unrelated dataset.

	Creative (N = 18)	Non-creative (N = 108)	Chr region	Chr: start-end	Event type	Genes	p-value
**Creative phenotype**	2 (11%)	0 (0%)	6q14.1	6: 76218758–76552776	loss	*MYO6.SENP6*	0.0194
	2 (11%)	0 (0%)	7q33	7: 133146663–133235334	loss	*EXOC4*	0.0194
	2 (11%)	0 (0%)	7q11.21	7: 64679561–65326821	loss	*INTS4L1.ZNF92*	0.0194
	3 (17%)	2 (2%)	12p11.21	12: 32004170–32056577	loss	*-*	0.0207
	0 (0%)	26 (24%)	3p12.3	3: 75419736–75655870	loss	*FAM86DP*	0.0232
	4 (22%)	5 (5%)	7p12.1	7: 52733291–52743803	gain	*-*	0.0237
	5 (28%)	9 (8%)	6p21.32	6: 32493790–32560859	loss	*-*	0.0298
	4 (22%)	6 (6%)	5p15.33	5: 674921–840717	loss	*ZDHHC11*	0.0359

Seven different CNVRs showed very suggestive association with the creative phenotype and one CNVR to the non-creative phenotype (Table 5). Of the mildly associated CNVRs, seven were deletions and one was duplication. Interestingly, the deletion at 5p15.33, for creative subjects was found in both the family-based (48%) and unrelated (22%) sample sets. The most promising, though preliminary, associations were seen between the creative phenotype and deletions at 6q14, 7q11 and 7q33 (Fisher p = 0.0194). 11% of creative vs. 0% of noncreative subjects were carrying each of these deletions. In previous studies, 6q14, containing the genes *MYO6* and *SEN6*, has been associated with intellectual disability and language delay [51]. The region 7q11, containing genes *INTS4L1* and *ZNF92*, is a candidate locus for epilepsy [52]. Interestingly, regions near to 7q33, containing the gene *EXOC4*, has previously been linked to speech and language disorders [53] and dyslexia [54]. Further, a deletion at 12p11 (17% creative vs. 2% non-creative), and a 70 kb deletion at 6p21 (28% creative vs. 8% non-creative) were weakly associated with creative functions in music (p = 0.0207 and p = 0.0298, respectively). A duplication at 7p12 was enriched in 22% of creative individuals. For the non-creative group, a deletion at 3p12, locating near to a susceptibility gene for reading and language disorder *ROBO1* [55], was more common compared to the creative group (24% non-creative vs. 0% creative).

We acknowledge that because of the multiple testing problem the associations detected here are only suggestive and preliminary.

## Discussion

### Low music test scores

In the genome wide analysis of CNVs in musical traits, several CNVRs containing genes that affect neurodevelopment, learning and memory were detected. The most relevant CNVR found here was a deletion at 5q31.1 covering the protocadherin-α gene cluster 1–9 (*Pcdha 1-9*). This deletion was found from both sample sets in subjects with low music test scores (COMB): 54% of the cases from two families (Table 3) and 7% from the unrelated subjects. There is an abundance of data about the neurophysiological effects of music perception and practice on the human brain [2], [3], [4], [5], [12]. However data on the effects at the cellular levels is so far missing. *Pcdha* is involved in neural migration, differentiation and synaptogenesis [56], [57], [58]. Katori and colleagues [60] discovered that Pcdhas are important in maturation of serotonergic projections in all or most of the brain regions. Previously, Fukuda et al. [59] showed abnormalities in *Pcdha* mutant mice in abilities important for musical aptitude, that is learning and memory. The human serotonin transporter gene (*SLC6A4*) together with arginine vasopressin receptor gene (*AVPR1A*) polymorphisms has been reported to associate with artistic creativity in professional dancers [50] and with short-term musical memory [19]. Based on the normal function of the brain, *Pcdha* may be a relevant candidate gene affecting music perception and practice. In our previous studies, *AVPR1A*, also related to learning and memory, was associated with musical aptitude and listening to music [16], [17].

Absolute pitch (AP), which is a rare ability to recognize the pitch of a musical tone without a reference pitch, was previously linked on chromosome 8q24.21 [13]. In our study, a 1.3 Mb long duplication was located in the core linkage region of AP (Figure 2) in a subject with low COMB scores. The overlapping region contains gene *ADCY8 (adenylate cyclase 8)* that is associated with synaptic plasticity, short-term memory performance [60] and with bipolar disorder [61]. Additionally, genes *GSDMC*, *FAM49B* and *ASAP1* lie in the region of duplication (RefSeq). Disorders caused by CNVs of dosage sensitive genes critical for the physiological function of nervous system have been identified [62]. Further, large duplications have shown harmful effects on neurodevelopment [43], [63]. Music test scores used in our study tests e.g. subject's ability to detect differences in pitch. Although there are no studies about the overlap of AP and the phenotypes studied here, it is intriguing to speculate whether the duplication in the AP region had a harmful effect on pitch perception accuracy in the multifaceted phenotype of musical aptitude.

### Creativity and psychiatric disorders

Creativity is an ability to produce work that is not only original but appropriate for the situation in which it occurs [64]. In our previous study creativity in music (here composing, improvising or arranging) showed substantial heritability [16]. Here, a duplication at 2p22.1, containing the *GALM* gene, was co-segregating with creative phenotype. *GALM* encodes galactose mutarotase which has been reported to increase serotonin release and membrane trafficking of the human serotonin transporter (5-HTT) [48]. *GALM* has been associated with serotonin transporter binding potential in the human thalamus [48], the region that is important for the music perception process [65]. Serotonin metabolism is disturbed in mood disorders e.g. depression [49]. Recently, serotonin transporter gene (*SLC6A4*) has been associated not only with psychiatric disorders but also with musical aptitude [16], attending choral singing [19] and creative dance performance [50]. It is known that genetic polymorphisms that are related to psychiatric disorders may have a positive impact on cognitive traits like creativity, IQ, and working memory [50], [66], [67], [68]. The other side of the coin is that highly creative individuals may have an elevated risk for certain types of psychiatric disorders. Kyaga et al. [68] suggested co-segregation of mental disorders with creativity in families. The risk alleles may act like plasticity genes, resulting in that carriers are more responsive to both positive and negative environmental experiences than others [69].

Music is non-verbal communication that is able to evoke emotions which are unique in intensity and state [70], [71]. Listening to and/or playing music is environmental stimuli that has multiple measurable effects on brain structure and function. Neurophysiological and brain imaging studies have discovered that music induces synaptic plasticity, e.g. active training and practicing music has been shown to enlarge cortical presentations in the somatosensory and auditory domains in professional musicians [72]. However, little is known about the effect of music on the brain function at the cellular level. In our study, a large 2.1 Mb long duplication in the region of the *MCTP2* gene, previously reported in schizophrenia [47] was co-segregated in three generations with low or average music test scores. *MCTP2* is involved in cellular signal transduction and synaptic functions by Ca^2+^ binding [73]. In schizophrenia, Ca^2+^ binding is altered in the prefrontal cortex [74]. This same brain region is also important for recognizing emotions in music [75], which makes us hypothesize the effect of this large duplication to the function of prefrontal cortex in music perception [41], [62], [63].

In our study, a deletion at 3p14.1 was ranked high in both family and unrelated data for different phenotypes; in the family data the non-creative phenotype, and in the unrelated sample set low music test scores. In our previous study high music test scores were associated with creativity in music (p<0.0001) [16]. This may support the finding that deletion at 3p14.1 is associated with both low music test scores and the non-creative phenotype.

### The size of the CNVRs

Currently, the standardized practices and gold standard for CNV studies, especially across different size spectrum, are lacking. Although several studies raised the issue of potential false discoveries among smaller CNVs (<100 kb), it remains intriguing for the researchers to study smaller CNVs because of their well-acknowledged functional impact [76], [77]. Moreover, CNVs>100 kb were previously suggested to have deleterious effects [23] and in general they have been heavily implicated in neurodevelopmental disorders. As this study focuses on a normal trait, our presumption allowed us to consider smaller CNVs also. However, we tried to minimize the false discoveries by using a multi-algorithm approach which has been promising in previous studies [39], [40], [41].

## Concluding Remarks

Here we report the results of the first genome wide CNV survey for music related phenotypes; musical aptitude and creative functions in music using both a family-based approach and case-control study. The advantages of this study are the use of both family-based and sporadic data. Moreover, all samples were genotyped at the same time using the same platform, increasing the reliability of the analyses. However, there are several limitations in our study. Definition of the phenotype here covers only a small portion of the multifaceted phenotype of music perception and practice. Being aware of the quantitative nature of musical aptitude, division of the phenotype to high COMB and low COMB groups is somewhat artificial. Also, the sample size is relatively small and the participants have not been screened for neurocognitive deficits. Consequently, the identified CNVs cannot be excluded here as being potentially predisposing for neuropsychiatric conditions. Because of the small sample size only suggestive associations were detected. The result, although interesting, is preliminary and replication with a larger sample set is needed.

The perception of sounds begins in the cochlea, the auditory portion of the inner ear, but the actual perceiving, processing and creating music takes place at multiple sites and elicits different functions of the brain [4], [5], [70], [78], [79], [80], [81]. The thalamus is responsible for projection of the sound information into the auditory cortex where more specific information about acoustic signal, like pitch height, chroma, intensity and timbre, is further extracted [4]. CNVs found in this study contain several genes that are expressed in brain regions where music is perceived, including hippocampus, thalamus and prefrontal cortex. Further studies are needed to survey the mechanisms of the detected CNVs and genes for the human brain and further to music perception and practice.

## Supporting Information

Table S1 All CNVRs ranked for COMB scores in the five study families.
(XLS)

Table S2 Rare CNV burden analysis by event type and size in High COMB VS low COMB subjects.
(DOCX)

Table S3 All CNVRs ranked for creative functions in music in the five study families.
(XLS)
