# Supplementary material for: Genome-Wide Copy Number Variation Analysis in Extended Families and Unrelated Individuals Characterized for Musical Aptitude and Creativity in Music
Source: PLoS One. 2013 Feb 27;8(2):e56356. doi: 10.1371/journal.pone.0056356 (PMC3584088; doi:10.1371/journal.pone.0056356)
Supplement: Table S2 — Rare CNV burden analysis by event type and size in High COMB VS low COMB subjects. (DOCX) [file pone.0056356.s002.docx]

Table S2.a Rare CNV burden analysis by event type and size in High COMB VS low COMB subjects

| Deletions (kb) | High COMB (n=43) | Low COMB (n=41) |
| --- | --- | --- |
| <10 | 104 | 109 |
| 10-50 | 68 | 84 |
| 50-100 | 12 | 14 |
| 100-400 | 22 | 12 |
| >400 | 3 | 1 |
| Duplications (kb) |  |  |
| <10 | 20 | 14 |
| 10-50 | 37 | 47 |
| 50-100 | 10 | 19 |
| 100-400 | 18 | 23 |
| >400 | 2 | 7 |
| Total (kb) |  |  |
| <10 | 124 | 123 |
| 10-50 | 105 | 131 |
| 50-100 | 22 | 33 |
| 100-400 | 40 | 35 |
| >400 | 5 | 8 |

Table S2.b Rare CNV burden analysis by event type and size in Creative VS Non-creative subjects

| Deletions (kb) | Creative (n=21) | Non-creative (n=125) |
| --- | --- | --- |
| <10 | 64 | 314 |
| 10-50 | 32 | 208 |
| 50-100 | 7 | 43 |
| 100-400 | 11 | 43 |
| >400 | 1 | 3 |
| Duplications (kb) |  |  |
| <10 | 12 | 46 |
| 10-50 | 19 | 145 |
| 50-100 | 9 | 43 |
| 100-400 | 16 | 48 |
| >400 | 2 | 15 |
| Total (kb) |  |  |
| <10 | 76 | 360 |
| 10-50 | 51 | 353 |
| 50-100 | 16 | 86 |
| 100-400 | 27 | 91 |
| >400 | 3 | 18 |

Table S2.c CNV burden analysis (all CNVs) by event type and size in High COMB VS low COMB subjects

| Deletions (kb) | High COMB (n=43) | Low COMB (n=41) |
| --- | --- | --- |
| <10 | 277 | 285 |
| 10-50 | 179 | 220 |
| 50-100 | 69 | 63 |
| 100-400 | 47 | 31 |
| >400 | 3 | 1 |
| Duplications (kb) |  |  |
| <10 | 53 | 48 |
| 10-50 | 83 | 86 |
| 50-100 | 32 | 49 |
| 100-400 | 48 | 42 |
| >400 | 2 | 7 |
| Total (kb) |  |  |
| <10 | 330 | 333 |
| 10-50 | 262 | 306 |
| 50-100 | 101 | 112 |
| 100-400 | 95 | 73 |
| >400 | 5 | 8 |

Table S2.d CNV burden analysis (all CNVs) by event type and size in Creative VS Non-creative subjects

| Deletions (kb) | Creative (n=21) | Non-creative (n=125) |
| --- | --- | --- |
| <10 | 146 | 837 |
| 10-50 | 91 | 558 |
| 50-100 | 31 | 171 |
| 100-400 | 19 | 111 |
| >400 | 1 | 3 |
| Duplications (kb) |  |  |
| <10 | 24 | 142 |
| 10-50 | 44 | 279 |
| 50-100 | 20 | 119 |
| 100-400 | 28 | 121 |
| >400 | 2 | 17 |
| Total (kb) |  |  |
| <10 | 170 | 979 |
| 10-50 | 135 | 837 |
| 50-100 | 51 | 290 |
| 100-400 | 47 | 232 |
| >400 | 3 | 20 |
